# Update: Characteristics of Symptomatic Women of Reproductive Age with Laboratory-Confirmed SARS-CoV-2 Infection by Pregnancy Status — United States, January 22–October 3, 2020

**DOI:** 10.15585/mmwr.mm6944e3

**Published:** 2020-11-06

**Authors:** Laura D. Zambrano, Sascha Ellington, Penelope Strid, Romeo R. Galang, Titilope Oduyebo, Van T. Tong, Kate R. Woodworth, John F. Nahabedian, Eduardo Azziz-Baumgartner, Suzanne M. Gilboa, Dana Meaney-Delman, CDC COVID-19 Response Pregnancy and Infant Linked Outcomes Team, CDC COVID-19 Response Pregnancy and Infant Linked Outcomes Team, Amanda Akosa, Carolyne Bennett, Veronica Burkel, Daniel Chang, Augustina Delaney, Charise Fox, Isabel Griffin, Jason Hsia, Katie Krause, Elizabeth Lewis, Susan Manning, Yousra Mohamoud, Suzanne Newton, Varsha Neelam, Emily O’Malley Olsen, Mirna Perez, Megan Reynolds, Aspen Riser, Maria Rivera, Nicole M. Roth, Christina Sancken, Neha Shinde, Ashley Smoots, Margaret Snead, Bailey Wallace, Florence Whitehill, Erin Whitehouse, Lauren Zapata

**Affiliations:** 1 CDC COVID-19 Response Team.; Eagle Global Scientific; Eagle Global Scientific; Eagle Medical; Oak Ridge Institute for Science and Education; CDC; Oak Ridge Institute for Science and Education; Eagle Global Scientific; CDC; CDC; CDC; CDC; CDC; CDC; CDC; CDC; CDC; CDC; CDC; CDC; Eagle Global Scientific; CDC; Eagle Global Scientific; CDC; CDC; CDC; Oak Ridge Institute for Science and Education; CDC; CDC

Studies suggest that pregnant women might be at increased risk for severe illness associated with coronavirus disease 2019 (COVID-19) (*1*,*2*). This report provides updated information about symptomatic women of reproductive age (15–44 years) with laboratory-confirmed infection with SARS-CoV-2, the virus that causes COVID-19. During January 22–October 3, CDC received reports through national COVID-19 case surveillance or through the National Notifiable Diseases Surveillance System (NNDSS) of 1,300,938 women aged 15–44 years with laboratory results indicative of acute infection with SARS-CoV-2. Data on pregnancy status were available for 461,825 (35.5%) women with laboratory-confirmed infection, 409,462 (88.7%) of whom were symptomatic. Among symptomatic women, 23,434 (5.7%) were reported to be pregnant. After adjusting for age, race/ethnicity, and underlying medical conditions, pregnant women were significantly more likely than were nonpregnant women to be admitted to an intensive care unit (ICU) (10.5 versus 3.9 per 1,000 cases; adjusted risk ratio [aRR] = 3.0; 95% confidence interval [CI] = 2.6–3.4), receive invasive ventilation (2.9 versus 1.1 per 1,000 cases; aRR = 2.9; 95% CI = 2.2–3.8), receive extracorporeal membrane oxygenation (ECMO) (0.7 versus 0.3 per 1,000 cases; aRR = 2.4; 95% CI = 1.5–4.0), and die (1.5 versus 1.2 per 1,000 cases; aRR = 1.7; 95% CI = 1.2–2.4). Stratifying these analyses by age and race/ethnicity highlighted disparities in risk by subgroup. Although the absolute risks for severe outcomes for women were low, pregnant women were at increased risk for severe COVID-19–associated illness. To reduce the risk for severe illness and death from COVID-19, pregnant women should be counseled about the importance of seeking prompt medical care if they have symptoms and measures to prevent SARS-CoV-2 infection should be strongly emphasized for pregnant women and their families during all medical encounters, including prenatal care visits. Understanding COVID-19–associated risks among pregnant women is important for prevention counseling and clinical care and treatment.

Data on laboratory-confirmed and probable COVID-19 cases^†^ were electronically reported to CDC using a standardized case report form^§^ or NNDSS^¶^ as part of COVID-19 surveillance efforts. Data are reported by health departments and can be updated by health departments as new information becomes available. This analysis included cases initially reported to CDC during January 22–October 3, 2020, with data updated as of October 28, 2020. Cases were limited to those in symptomatic women aged 15–44 years in the United States with laboratory-confirmed infection (detection of SARS-CoV-2 RNA in a clinical specimen using a molecular amplification detection test). Information on demographic characteristics, pregnancy status, underlying medical conditions, symptoms, and outcomes was collected. Pregnancy status was ascertained by a pregnancy field on the COVID-19 case report form or through records linked to the Surveillance for Emerging Threats to Mothers and Babies Network (SET-NET) optional COVID-19 module**^,^^††^ (*3*). CDC ascertained symptom status either through a reported symptom status variable (symptomatic, asymptomatic, or unknown) or based on the presence of at least one specific symptom on the case report form. Outcomes with missing data were assumed not to have occurred. Crude and adjusted RRs and 95% CIs were calculated using modified Poisson regression. Overall and stratified risk ratios were adjusted for age (in years), race/ethnicity, and presence of diabetes, cardiovascular disease (including hypertension), and chronic lung disease. SAS (version 9.4; SAS Institute) was used to conduct all analyses. This activity was reviewed by CDC and was conducted consistent with applicable federal law and CDC policy.^§§^

During January 22–October 3, a total of 5,003,041 laboratory-confirmed cases of SARS-CoV-2 infection were reported to CDC as part of national COVID-19 case surveillance, including 1,300,938 (26.0%) cases in women aged 15–44 years. Data on pregnancy status were available for 461,825 (35.5%) women aged 15–44 years, 30,415 (6.6%) of whom were pregnant and 431,410 (93.4%) of whom were nonpregnant. Among all women aged 15–44 years with known pregnancy status, 409,462 (88.7%) were symptomatic, including 23,434 pregnant women, accounting for 5.7% of all symptomatic women with laboratory-confirmed COVID-19, and 386,028 nonpregnant women. Pregnant women were more frequently Hispanic/Latina (Hispanic) (29.7%) and less frequently non-Hispanic White (White) (23.5%) compared with nonpregnant women (22.6% Hispanic and 31.7% White). Among all women, cough, headache, muscle aches, and fever were the most frequently reported signs and symptoms; most symptoms were reported less frequently by pregnant women than by nonpregnant women (Table 1).

**TABLE 1:** Demographic characteristics, signs and symptoms, and underlying medical conditions among symptomatic women of reproductive age with laboratory-confirmed SARS-CoV-2 infection (N = 409,462),*^,†^ by pregnancy status — United States, January 22–October 3, 2020

Characteristic	No. (%) of symptomatic women		
	Pregnant (n = 23,434)	Nonpregnant (n = 386,028)	Total (N = 409,462)
**Age group, yrs**			
15–24	6,463 (27.6)	133,032 (34.5)	139,495 (34.1)
25–34	12,951 (55.3)	131,835 (34.2)	144,786 (35.4)
35–44	4,020 (17.2)	121,161 (31.4)	125,181 (30.6)
**Race/Ethnicity** ^§^			
Hispanic or Latina, any race	6,962 (29.7)	85,618 (22.2)	92,580 (22.6)
AI/AN, non-Hispanic	113 (0.5)	1,652 (0.4)	1,765 (0.4)
Asian, non-Hispanic	560 (2.4)	8,605 (2.2)	9,165 (2.2)
Black, non-Hispanic	3,387 (14.5)	54,185 (14.0)	57,572 (14.1)
NHPI, non-Hispanic	119 (0.5)	1,526 (0.4)	1,645 (0.4)
White, non-Hispanic	5,508 (23.5)	124,305 (32.2)	129,813 (31.7)
Multiple or other race, non-Hispanic	726 (3.1)	12,341 (3.2)	13,067 (3.2)
**Signs and symptoms**			
Known status of individual signs and symptoms^¶^	10,404	174,198	184,602
Cough	5,230 (50.3)	89,422 (51.3)	94,652 (51.3)
Fever**	3,328 (32.0)	68,536 (39.3)	71,864 (38.9)
Muscle aches	3,818 (36.7)	78,725 (45.2)	82,543 (44.7)
Chills	2,537 (24.4)	50,836 (29.2)	53,373 (28.9)
Headache	4,447 (42.7)	95,713 (54.9)	100,160 (54.3)
Shortness of breath	2,692 (25.9)	43,234 (24.8)	45,926 (24.9)
Sore throat	2,955 (28.4)	60,218 (34.6)	63,173 (34.2)
Diarrhea	1,479 (14.2)	38,165 (21.9)	39,644 (21.5)
Nausea or vomiting	2,052 (19.7)	28,999 (16.6)	31,051 (16.8)
Abdominal pain	870 (8.4)	16,123 (9.3)	16,993 (9.2)
Runny nose	1,328 (12.8)	22,750 (13.1)	24,078 (13.0)
New loss of taste or smell^††^	2,234 (21.5)	43,256 (24.8)	45,490 (24.6)
Fatigue	1,404 (13.5)	29,788 (17.1)	31,192 (16.9)
Wheezing	172 (1.7)	3,743 (2.1)	3,915 (2.1)
Chest pain	369 (3.5)	7,079 (4.1)	7,448 (4.0)
**Underlying medical conditions**			
Known underlying medical condition status^§§^	7,795	160,065	167,860
Diabetes mellitus	427 (5.5)	6,119 (3.8)	6,546 (3.9)
Cardiovascular disease	304 (3.9)	7,703 (4.8)	8,007 (4.8)
Chronic lung disease	506 (6.5)	9,185 (5.7)	9,691 (5.8)
Chronic renal disease	18 (0.2)	680 (0.4)	698 (0.4)
Chronic liver disease	17 (0.2)	350 (0.2)	367 (0.2)
Immunocompromised condition	124 (1.6)	2,496 (1.6)	2,620 (1.6)
Neurologic disorder, neurodevelopmental disorder, or intellectual disability	44 (0.6)	1,097 (0.7)	1,141 (0.7)
Psychiatric disorder	62 (0.8)	1,139 (0.7)	1,201 (0.7)
Autoimmune disorder	26 (0.3)	515 (0.3)	541 (0.3)
Severe obesity^¶¶^	174 (2.2)	1,810 (1.1)	1,984 (1.2)

**Abbreviations:** AI/AN = American Indian or Alaska Native; NHPI = Native Hawaiian or Other Pacific Islander.

* Women with known pregnancy status, representing 52% of 783,072 total cases among symptomatic women aged 15–44 years.

**^†^** All statistical comparisons were significant at α <0.01, with the exception of the comparison of prevalence of neurologic disorders between pregnant and nonpregnant women (p = 0.307).

^§^ Race/ethnicity was missing for 6,059 (26%) of symptomatic pregnant women and 97,796 (26%) of symptomatic nonpregnant women.

^¶^ Data on individual symptoms were known for 10,404 (44%) of pregnant women and 174,198 (45%) of nonpregnant women. Individual symptoms were considered known if any of the following symptoms were noted as present or absent on the CDC’s Human Infection with 2019 Novel Coronavirus Case Report Form: fever (measured >100.4°F [38°C] or subjective), cough, shortness of breath, wheezing, difficulty breathing, chills, rigors, myalgia, rhinorrhea, sore throat, chest pain, nausea or vomiting, abdominal pain, headache, fatigue, diarrhea (three or more loose stools in a 24-hour period), new olfactory or taste disorder, or other symptom not otherwise specified on the form.

** Patients were included if they had information for either measured or subjective fever variables and were considered to have a fever if “yes” was indicated for either variable.

^††^ New olfactory and taste disorder has only been included on the CDC’s Human Infection with 2019 Novel Coronavirus Case Report Form since May 5, 2020. Therefore, data might be underreported for this symptom.

^§§^ Status was classified as “known” if any of the following conditions were noted as present or absent on the CDC’s Human Infection with 2019 Novel Coronavirus Case Report Form: diabetes mellitus, cardiovascular disease (including hypertension), severe obesity (body mass index ≥40 kg/m^2^), chronic renal disease, chronic liver disease, chronic lung disease, immunosuppressive condition, autoimmune condition, neurologic condition (including neurodevelopmental, intellectual, physical, visual, or hearing impairment), psychological/psychiatric condition, and other underlying medical condition not otherwise specified.

^¶¶^ Defined as body mass index ≥40 kg/m^2^.

Compared with nonpregnant women, pregnant women more frequently were admitted to an ICU (10.5 versus 3.9 per 1,000 cases; aRR = 3.0; 95% CI = 2.6–3.4), received invasive ventilation (2.9 versus 1.1 per 1,000 cases; aRR = 2.9; 95% CI = 2.2–3.8) and received ECMO (0.7 versus 0.3 per 1,000 cases; aRR = 2.4; 95% CI = 1.5–4.0). Thirty-four deaths (1.5 per 1,000 cases) were reported among 23,434 symptomatic pregnant women, and 447 (1.2 per 1,000 cases) were reported among 386,028 nonpregnant women, reflecting a 70% increased risk for death associated with pregnancy (aRR = 1.7; 95% CI = 1.2–2.4). Irrespective of pregnancy status, ICU admissions, receipt of invasive ventilation, and death occurred more often among women aged 35–44 years than among those aged 15–24 years (Table 2). Whereas non-Hispanic Black or African American (Black) women made up 14.1% of women included in this analysis, they represented 176 (36.6%) deaths overall, including nine of 34 (26.5%) deaths among pregnant women and 167 of 447 (37.4%) deaths among nonpregnant women.

**TABLE 2:** Intensive care unit (ICU) admissions, receipt of invasive ventilation, receipt of extracorporeal membrane oxygenation (ECMO), and deaths among symptomatic women of reproductive age with laboratory-confirmed SARS-CoV-2 (N = 409,462), by pregnancy status, age, race/ethnicity, and underlying health conditions — United States, January 22–October 3, 2020

Outcome*/Characteristic	No. (per 1,000 cases) of symptomatic women		Risk ratio (95% CI)	
	Pregnant (n = 23,434)	Nonpregnant (n = 386,028)	Crude^†^	Adjusted^†,§^
**ICU admission^¶^**				
**All**	**245 (10.5)**	**1,492 (3.9)**	**2.7 (2.4–3.1)**	**3.0 (2.6–3.4)**
**Age group, yrs**				
15–24	49 (7.6)	244 (1.8)	4.1 (3.0–5.6)	3.9 (2.8–5.3)
25–34	118 (9.1)	467 (3.5)	2.6 (2.1–3.1)	2.4 (2.0–3.0)
35–44	78 (19.4)	781 (6.4)	3.0 (2.4–3.8)	3.2 (2.5–4.0)
**Race/Ethnicity**				
Hispanic or Latina	89 (12.8)	429 (5.0)	2.6 (2.0–3.2)	2.8 (2.2–3.5)
AI/AN, non-Hispanic	0 (0)	13 (7.9)	NA	NA
Asian, non-Hispanic	20 (35.7)	52 (6.0)	5.9 (3.6–9.8)	6.6 (4.0–11.0)
Black, non-Hispanic	46 (13.6)	334 (6.2)	2.2 (1.6–3.0)	2.8 (2.0–3.8)
NHPI, non-Hispanic	5 (42.0)	22 (14.4)	2.9 (1.1–7.6)	3.7 (1.3–10.1)
White, non-Hispanic	31 (5.6)	348 (2.8)	2.0 (1.4–2.9)	2.3 (1.6–3.3)
Multiple or other race, non-Hispanic	8 (11.0)	37 (3.0)	3.7 (1.7–7.9)	4.1 (1.9–8.9)
Unknown/Not reported	46 (7.6)	257 (2.6)	2.9 (2.1–3.9)	3.4 (2.5–4.7)
**Underlying health conditions**				
Diabetes	25 (58.5)	274 (44.8)	1.3 (0.9–1.9)	1.5 (1.0–2.2)
CVD**	13 (42.8)	247 (32.1)	1.3 (0.8–2.3)	1.5 (0.9–2.6)
Chronic lung disease	15 (29.6)	179 (19.5)	1.5 (0.9–2.6)	1.7 (1.0–2.8)
**Invasive ventilation^††^**				
**All**	**67 (2.9)**	**412 (1.1)**	**2.7 (2.1–3.5)**	**2.9 (2.2–3.8)**
**Age group, yrs**				
15–24	11 (1.7)	68 (0.5)	3.3 (1.8–6.3)	3.0 (1.6–5.7)**^§§^**
25–34	30 (2.3)	123 (0.9)	2.5 (1.7–3.7)	2.5 (1.6–3.7)**^§§^**
35–44	26 (6.5)	221 (1.8)	3.5 (2.4–5.3)	3.6 (2.4–5.4)
**Race/Ethnicity**				
Hispanic or Latina	33 (4.7)	143 (1.7)	2.8 (1.9–4.1)	3.0 (2.1–4.5)
AI/AN, non-Hispanic	0 (0)	5 (3.0)	NA	NA
Asian, non-Hispanic	4 (7.1)	19 (2.2)	NA	NA
Black, non-Hispanic	10 (3)	86 (1.6)	1.9 (1.0–3.6)	2.5 (1.3–4.9)
NHPI, non-Hispanic	4 (33.6)	10 (6.6)	NA	NA
White, non-Hispanic	12 (2.2)	102 (0.8)	2.7 (1.5–4.8)	3.0 (1.7–5.6)
Multiple or other race, non-Hispanic	0 (0)	8 (0.6)	NA	NA
Unknown/Not reported	4 (0.7)	39 (0.4)	NA	NA
**Underlying health conditions**				
Diabetes	10 (23.4)	98 (16.0)	1.5 (0.8–2.8)	1.7 (0.9–3.3)
CVD**	6 (19.7)	82 (10.6)	1.9 (0.8–4.2)	1.9 (0.8–4.5)**^¶¶^**
Chronic lung disease	4 (7.9)	50 (5.4)	NA	NA
ECMO***				
**All**	**17 (0.7)**	**120 (0.3)**	**2.3 (1.4–3.9)**	**2.4 (1.5–4.0)**
**Age group,yrs**				
15–24	6 (0.9)	31 (0.2)	4.0 (1.7–9.5)	NA^†††^
25–34	7 (0.5)	35 (0.3)	2.0 (0.9–4.6)	2.0 (0.9–4.4)**^§§^**
35–44	4 (1.0)	54 (0.4)	NA	NA
**Race/Ethnicity**				
Hispanic or Latina	6 (0.9)	35 (0.4)	2.1 (0.9–5.0)	2.4 (1.0–5.9)
AI/AN, non-Hispanic	0 (0)	1 (0.6)	NA	NA
Asian, non-Hispanic	0 (0)	1 (0.1)	NA	NA
Black, non-Hispanic	5 (1.5)	30 (0.6)	2.7 (1.0–6.9)	2.9 (1.1–7.3)
NHPI, non-Hispanic	0 (0)	2 (1.3)	NA	NA
White, non-Hispanic	4 (0.7)	29 (0.2)	NA	NA
Multiple or other race, non-Hispanic	0 (0)	3 (0.2)	NA	NA
Unknown/Not reported	2 (0.3)	19 (0.2)	NA	NA
**Underlying health conditions**				
Diabetes	1 (2.3)	13 (2.1)	NA	NA
CVD**	1 (3.3)	20 (2.6)	NA	NA
Chronic lung disease	1 (2.0)	20 (2.2)	NA	NA
**Death^§§§^**				
**All**	**34 (1.5)**	**447 (1.2)**	**1.3 (0.9–1.8)**	**1.7 (1.2–2.4)**
**Age group, yrs**				
15–24	2 (0.3)	40 (0.3)	NA	NA
25–34	15 (1.2)	125 (0.9)	1.2 (0.7–2.1)	1.2 (0.7–2.1)
35–44	17 (4.2)	282 (2.3)	1.8 (1.1–3.0)	2.0 (1.2–3.2)
**Race/Ethnicity**				
Hispanic or Latina	14 (2.0)	87 (1.0)	2.0 (1.1–3.5)	2.4 (1.3–4.3)
AI/AN, non-Hispanic	0 (0)	5 (3.0)	NA	NA
Asian, non-Hispanic	1 (1.8)	11 (1.3)	NA	NA
Black, non-Hispanic	9 (2.7)	167 (3.1)	0.9 (0.4–1.7)	1.4 (0.7–2.7)
NHPI, non-Hispanic	2 (16.8)	6 (3.9)	NA	NA
White, non-Hispanic	3 (0.5)	83 (0.7)	NA	NA
Multiple or other race, non-Hispanic	0 (0)	12 (1.0)	NA	NA
Unknown/Not reported	5 (0.8)	76 (0.8)	1.1 (0.4–2.6)	1.4 (0.6–3.6)
**Underlying health conditions**				
Diabetes	6 (14.1)	78 (12.7)	1.1 (0.5–2.5)	1.5 (0.6–3.5)**^¶¶¶^**
CVD**	7 (23.0)	89 (11.6)	2.0 (0.9–4.3)	2.2 (1.0–4.8)****
Chronic lung disease	1 (2.0)	37 (4.0)	NA	NA

**Abbreviations:** AI/AN = American Indian/Alaska Native; CI = confidence interval; CVD = cardiovascular disease; NA = not applicable; NHPI = Native Hawaiian or Other Pacific Islander.

* Percentages calculated among total in pregnancy status group.

^†^ Crude and adjusted risk ratios were not calculated for cell sizes <5.

^§^ Adjusted for age (continuous variable, in years), categorical race/ethnicity variable, and dichotomous indicators for diabetes, cardiovascular disease, and chronic lung disease.

^¶^ A total of 17,007 (72.6%) symptomatic pregnant women and 291,539 (75.5%) symptomatic nonpregnant women were missing information on ICU admission status; however, while hospital admission status was not separately analyzed, hospitalization status was missing for 2,393 (10.2%) symptomatic pregnant women and 35,624 (9.2%) of symptomatic nonpregnant women, and no hospital admission was reported for 16,672 (71.1%) pregnant and 337,414 (87.4%) nonpregnant women. Therefore, in the absence of reported hospital admissions, women with missing ICU admission information were assumed to have not been admitted to the ICU.

** Cardiovascular disease also accounts for presence of hypertension.

^††^ A total of 17,903 (76.4%) pregnant women and 299,413 (77.6%) nonpregnant women were missing information regarding receipt of invasive ventilation and were assumed to have not received it.

**^§§^** Adjusted for the presence of diabetes, CVD, and chronic lung disease only, and removed race/ethnicity from adjustment set because of model convergence issues**^.^**

**^¶¶^** Adjusted for the presence of diabetes and chronic lung disease and age as a continuous covariate only and removed race/ethnicity from adjustment set because of model convergence issues.

*** A total of 18,246 (77.9%) pregnant women and 298,608 (77.4%) nonpregnant women were missing information for receipt of ECMO and were assumed to have not received ECMO.

^†††^ Model failed to converge even after adjustment for a reduced set of covariates.

^§§§^ A total of 5,152 (22.0%) pregnant women and 66,346 (17.2%) nonpregnant women were missing information on death and were assumed to have survived.

**^¶¶¶^** Adjusted for the presence of CVD and chronic lung disease and age as a continuous variable.

**** Adjusted for presence of diabetes and chronic lung disease and age as a continuous variable.

Increased risk for ICU admission among pregnant women was observed for all strata but was particularly notable among non-Hispanic Asian (Asian) women (aRR = 6.6; 95% CI = 4.0–11.0) and non-Hispanic Native Hawaiian/Pacific Islander women (aRR = 3.7; 95% CI = 1.3–10.1). Risk for receiving invasive ventilation among pregnant women aged 15–24 years was 3.0 times that of nonpregnant women (95% CI = 1.6–5.7), and among pregnant women aged 35–44 years was 3.6 times that of nonpregnant women (95% CI = 2.4–5.4). In addition, among Hispanic women, pregnancy was associated with 2.4 times the risk for death (95% CI = 1.3-4.3) (Table 2). 

## Discussion

Although the absolute risks for severe COVID-19–associated outcomes among women were low, pregnant women were at significantly higher risk for severe outcomes compared with nonpregnant women. This finding might be related to physiologic changes in pregnancy, including increased heart rate and oxygen consumption, decreased lung capacity, a shift away from cell-mediated immunity, and increased risk for thromboembolic disease (*4*,*5*). Compared with the initial report of these data (*1*), in which increased risk for ICU admissions and invasive ventilation among pregnant women was reported, this analysis includes nearly five times the number of symptomatic women and a higher proportion of women with known pregnancy status (36% versus 28%). Further, to avoid including pregnant women who were tested as part of asymptomatic screening practices at the delivery hospitalization, this analysis was limited to symptomatic women. In this analysis 5.7% of symptomatic women aged 15–44 years with COVID-19 were pregnant, corresponding to the anticipated proportion of 5% of the population at any point in time.^¶¶^^,^***

Whereas increased risk for severe disease related to pregnancy was apparent in nearly all stratified analyses, pregnant women aged 35–44 years with COVID-19 were nearly four times as likely to require invasive ventilation and twice as likely to die than were nonpregnant women of the same age. Among symptomatic pregnant women with COVID-19 for whom race/ethnicity was reported, 30% were Hispanic and 24% were White, differing from the overall reported racial/ethnic distribution of women who gave birth in 2019 (24% Hispanic and 51% White).^†††^ Pregnant Asian and Native Hawaiian/Pacific Islander women appeared to be at disproportionately greater risk for ICU admission. Hispanic pregnant women of any race not only experienced a disproportionate risk for SARS-CoV-2 infection but also a higher risk for death compared with nonpregnant Hispanic women. Regardless of pregnancy status, non-Hispanic Black women experienced a disproportionate number of deaths relative to their distribution among reported cases. This analysis highlights racial and ethnic disparities in both risk for infection and disease severity among pregnant women, indicating a need to address potential drivers of risk in these populations.

The findings in this report are subject to at least three limitations. First, national case surveillance data for COVID-19 are voluntarily reported to CDC and rely on health care providers and jurisdictional public health agencies to share information for patients who meet standard case definitions. The mechanism used to report cases and the capacity to investigate cases varies across jurisdictions.^§§§^ Thus, case information is limited or unavailable for a portion of detected COVID-19 cases, and reported case data might be updated at any time. This analysis was restricted to women with known age; however, pregnancy status was missing for over one half (64.5%) of reported cases, and among those with known pregnancy status, data on race/ethnicity were missing for approximately 25% of cases, and information on symptoms and underlying conditions was missing for approximately one half. Second, when estimating the proportion of cases with severe outcomes, the observational data collected through passive surveillance might be subject to reporting bias, wherein preferential ascertainment of severe cases is likely (*6*,*7*); therefore, the frequency of reported outcomes incorporates a denominator of all cases as a conservative estimate. Finally, severe outcomes might require additional time to be ascertained. To account for this, a time lag was incorporated, such that data reported as of October 28, 2020, were used for cases reported as of October 3.

This analysis supports previous findings that pregnancy is associated with increased risk for ICU admission and receipt of invasive ventilation among women of reproductive age with COVID-19 (*1**,**2*). In the current report, an increased risk for receiving ECMO and death was also observed, which are two additional important markers of COVID-19 severity that support previous findings. In comparison to influenza, a recent meta-analysis found no increased risk for ICU admission or death among pregnant women with seasonal influenza (*8*). However, data from previous influenza pandemics, including 2009 H1N1, have shown that pregnant women are at increased risk for severe outcomes including death and the absolute risks for severe outcomes were higher than in this study of COVID-19 during pregnancy (*9**)*. Longitudinal surveillance and cohort studies among pregnant women with COVID-19, including information about pregnancy outcomes, are necessary to understand the full spectrum of maternal and neonatal outcomes associated with COVID-19 in pregnancy. CDC, in collaboration with health departments, has adapted SET-NET to collect pregnancy-related information and pregnancy and neonatal outcomes among women with COVID-19 during pregnancy^¶¶¶^ (*3*).

Understanding the risk posed by SARS-CoV-2 infection in pregnant women can inform clinical practice, risk communication, and medical countermeasure allocation. Pregnant women should be informed of their risk for severe COVID-19–associated illness and the warning signs of severe COVID-19.**** To minimize the risk for acquiring SARS-CoV-2 infection, pregnant women should limit unnecessary interactions with persons who might have been exposed to or are infected with SARS-CoV-2, including those within their household,^††††^ as much as possible.^§§§§^ When going out or interacting with others, pregnant women should wear a mask, social distance, avoid persons who are not wearing a mask, and frequently wash their hands. In addition, pregnant women should take measures to ensure their general health, including staying up to date with annual influenza vaccination and prenatal care. Providers who care for pregnant women should be familiar with guidelines for medical management of COVID-19, including considerations for management of COVID-19 in pregnancy.^¶¶¶¶^^,^***** Additional data from surveillance and cohort studies on COVID-19 severity during pregnancy are necessary to inform messaging and patient counseling.

**Summary**

**What is already known about this topic?**

Limited information suggests that pregnant women with COVID-19 might be at increased risk for severe illness compared with nonpregnant women.

**What is added by this report?**

In an analysis of approximately 400,000 women aged 15–44 years with symptomatic COVID-19, intensive care unit admission, invasive ventilation, extracorporeal membrane oxygenation, and death were more likely in pregnant women than in nonpregnant women.

**What are the implications for public health practice?**

Pregnant women should be counseled about the risk for severe COVID-19–associated illness including death; measures to prevent infection with SARS-CoV-2 should be emphasized for pregnant women and their families. These findings can inform clinical practice, risk communication, and medical countermeasure allocation.

## Footnotes

* These authors contributed equally to this report.

†
https://wwwn.cdc.gov/nndss/conditions/coronavirus-disease-2019-covid-19/case-definition/2020/08/05/ .

§
https://www.cdc.gov/coronavirus/2019-ncov/downloads/pui-form.pdf .

¶
https://wwwn.cdc.gov/nndss/covid-19-response.html .

**
https://www.cdc.gov/coronavirus/2019-ncov/downloads/cases-updates/case-report-form-pregnancy-module.pdf .

†† https://www.researchsquare.com/article/rs-90329/v1.

§§ 45 C.F.R. part 46, 21 C.F.R. part 56; 42 U.S.C. Sect. 241(d); 5 U.S.C. Sect. 552a; 44 U.S.C. Sect. 3501 et seq.

¶¶ https://data.census.gov/cedsci/table?q=United%20States&tid=ACSST1Y2019.S0101&hidePreview=false.

***
https://www.cdc.gov/reproductivehealth/emergency/docs/Geographic-Calculator-for-Pregnant-Women_508.xlsx .

†††
https://www.cdc.gov/nchs/data/vsrr/vsrr-8-508.pdf .

§§§ https://www.cdc.gov/coronavirus/2019-ncov/covid-data/faq-surveillance.html.

¶¶¶
https://www.cdc.gov/ncbddd/aboutus/pregnancy/emerging-threats.html .

****
https://www.cdc.gov/coronavirus/2019-ncov/symptoms-testing/symptoms.html .

†††† https://www.cdc.gov/coronavirus/2019-ncov/if-you-are-sick/care-for-someone.html#face-covering.

§§§§
https://www.cdc.gov/coronavirus/2019-ncov/need-extra-precautions/pregnancy-breastfeeding.html .

¶¶¶¶
https://www.covid19treatmentguidelines.nih.gov/ .

*****
https://www.acog.org/en/Topics/COVID-19 .
